# Paraneoplastic Limbic Encephalitis Resembling Acute Herpetic Encephalitis

**DOI:** 10.1155/2013/608643

**Published:** 2013-11-27

**Authors:** Ioannis Markakis, Athanasios Papathanasiou, Ermioni Papageorgiou, Kostantinos Siarkos, Georgios Gkekas

**Affiliations:** ^1^Department of Neurology, “St. Panteleimon” General State Hospital of Piraeus, 18454 Nikaia, Greece; ^2^Department of Neurology, Essex Centre for Neurological Sciences, Queen's Hospital, Romford, Essex RM7 0AG, UK

## Abstract

*Introduction*. Paraneoplastic limbic encephalitis (PLE) is a rare disorder that typically follows a chronic or subacute course of personality changes, memory loss, seizures, and hallucinations. Early diagnosis is difficult and characteristic symptoms can be mimicked by a variety of conditions. We present a case of PLE, initially presenting as acute herpetic encephalitis. *Case Presentation*. A 56-year-old male was admitted for evaluation of acute onset headache, fever, and confusion. On neurological examination he was confused with MMSE score of 15/30. CSF analysis revealed marked lymphocytic pleocytosis. A possible diagnosis of acute herpetic encephalitis was rendered and patient was treated with acyclovir. CSF PCR was negative. Cranial MRI revealed bilateral hyperintense lesions in medial temporal lobes with contrast enhancement. Despite treatment with acyclovir patient was deteriorated; thus, a paraneoplastic syndrome was suspected. Chest CT showed a right paratracheal lymph node mass, while a biopsy revealed neuroendocrine lung cancer. Auto antibodies to Hu were also detected. The patient was treated with steroids and chemotherapy. Six months later, he had complete tumour remission and marked neurological improvement. *Discussion*. PLE can rarely invade acutely, being indistinguishable from herpetic encephalitis. Inclusion of PLE in the differential diagnosis of acute encephalitis is of great clinical significance.

## 1. Introduction

Limbic encephalitis (LE) is a rather rare disorder that mainly affects limbic structures and is characterized by mood-personality changes, sleep disturbances, seizures, hallucinations, and short-term memory loss that can progress to dementia. In most patients with typical LE, the diagnosis is suggested by the clinical presentation, combined with EEG findings (epileptic activity in one or both temporal lobes and focal or generalized slow activity), MRI (hyperintense signals in the medial portion of one or both temporal lobes), and the indicated CSF inflammatory changes. Although nonparaneoplastic and paraneoplastic limbic encephalitis (PLE) have similar clinical features, identification of the paraneoplastic cause commonly depends on finding the tumour, the paraneoplastic antibodies, or both [1].

PLE results from production of a neuronal protein by a tumour, which precipitates an immune-mediated reaction (humoral and T-cell mediated) against both the tumour and the central nervous system itself. There are two types of PLE, one with antibodies to intracellular antigens such as Hu, Ma2, CRMP5, and amphiphysin, that is considered to be T-cell mediated, and LE with antibodies to cell-membrane antigens such as LGI1, CASPR2, NMDA, AMPA, and GABA. These antibodies are more likely directly involved in pathogenesis; thus, these forms of LE are more responsive to immune-based treatment. Although they are usually non-paraneoplastic, there is a variable percentage of an associating tumour [1]. PLE complicates several types of cancer, mainly small-cell lung carcinoma, testicular germ-cell neoplasms, breast cancer, thymoma, Hodgkin's lymphoma, or teratoma. PLE typically follows a subacute or chronic clinical course, with progression of symptoms within weeks to months [1–3].

Herein, we report a patient with PLE, initially presenting as acute herpetic encephalitis.

## 2. Case Presentation

A 56-year-old male was admitted for evaluation of headache, fever (up to 38°C), and acute confusional state since two days. His past medical history was remarkable for arterial hypertension on perindopril 4 mg od and heavy smoking (40 cig/day). There were no neurologically affected family members. On neurological examination he was alert but confused with a Mini Mental Status Examination (MMSE) score of 15/30 (orientation to time 1/5, orientation to place 1/5, registration 3/3, attention and calculation 1/5, recall 0/3, language and complex commands 8/8, and construction 1/1). There were no meningeal or pyramidal signs, cranial nerves were intact and there was no evidence of sensory dysfunction.

**Figure 1:**
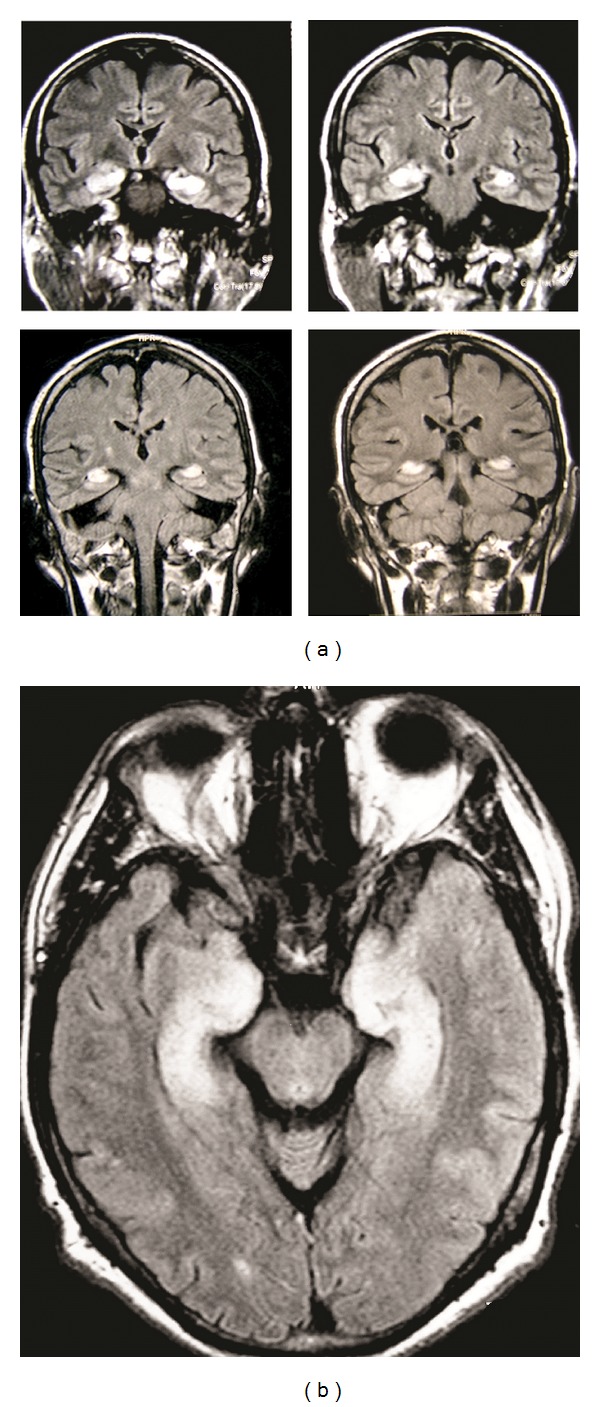
(a) Coronal FLAIR brain MR images demonstrate hyperintense lesions in medial temporal lobes bilaterally. (b) Axial FLAIR brain MR image demonstrates hyperintense lesions in medial temporal lobes bilaterally.

**Figure 2:**
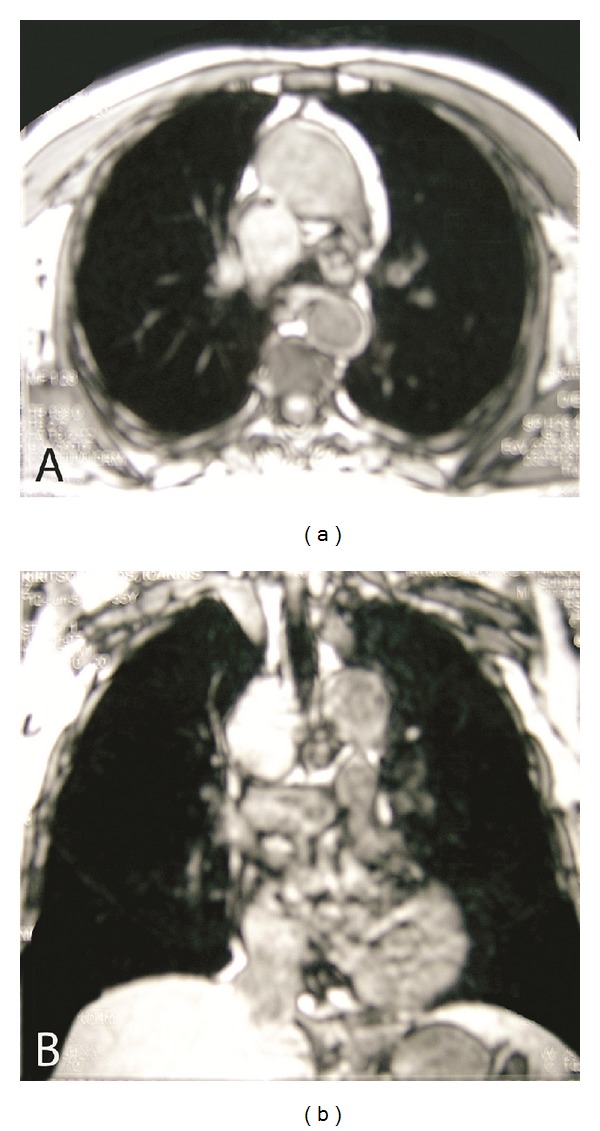
Axial (a) and coronal (b) chest MR images demonstrate right paratracheal lymph node mass.

Brain CT was normal, while CSF analysis revealed marked pleocytosis (170 cells/mm^3^; lymphocytes: 90%), increased protein (120 mg/dL) and normal glucose (71 mg/dL, serum: 110 mg/dL). EEG showed diffuse slow activity with paroxysmal slow wave bursts. Brain MRI revealed bilateral enhancing T2 hyperintense lesions in medial temporal lobes (Figures 1(a) and 1(b)). A working diagnosis of acute herpetic encephalitis was rendered and patient was treated with intravenous acyclovir and levetiracetam. Routine laboratory assays were normal. Serological tests and CSF polymerase chain reaction for infectious pathogens (HSV1/2, VZV, CMV, HHV-6, and Treponema pallidum) were negative. On the third day of his hospitalization, the patient developed aphasia, agitation, and irritability, accompanied by two generalized tonic-clonic seizures that were treated with valproic acid. Six days later, he became afebrile but his cognitive function continued to decline, despite treatment with intravenous acyclovir. A new lumbar puncture was performed that revealed normal cell count and protein, with positive oligoclonal bands. A paraneoplastic syndrome was suspected; thus, chest-abdomen CT and full gastrointestinal endoscopy were performed. Chest CT showed a right paratracheal lymph node mass that was confirmed with chest MRI (Figure 2). In addition, serological tests for onconeuronal antibodies revealed autoantibodies to Hu. Thoracic surgeon consultation recommended a thoracotomy, where a biopsy demonstrated a large cell neuroendocrine carcinoma. The patient was treated with intravenous corticosteroids, carboplatin, and etoposide and was discharged with 40 mg prednisolone od, 500 mg valproic acid tds, 500 mg levetiracetam bd, and 25 mgr quetiapine at night. Six months later, he had complete tumour remission as demonstrated in chest CT, MRI lesions have been resolved, and the patient was fully functional with a MMSE score of 30/30.

## 3. Discussion

Herein we report a patient that developed fever, confusion, anterograde amnesia, and seizures within a few days. MRI revealed bilateral enhancing lesions in medial temporal lobes, compatible with LE. LE encompasses a group of clinicopathological entities that affect the medial temporal lobes and other limbic structures (cingulate gyrus, orbital cortex, and hypothalamus), leading to personality changes, seizures, alterations of consciousness and anterograde amnesia. MRI is crucial in the diagnosis of these disorders, revealing characteristic mediotemporal lesions which may or may not enhance after contrast administration. The differential diagnosis varies widely and includes infectious causes, paraneoplastic disorders, Hashimoto's encephalopathy, systemic lupus erythematosus, Sjogren's syndrome, toxic-metabolic encephalopathy (including Korsakoff's syndrome), primary angiitis of CNS, syphilis, brain metastasis, low grade glioma, and gliomatosis cerebri [1]. Full laboratory assays excluded most of the above mentioned causes, while CSF analysis demonstrated an intense lymphocytic pleocytosis. Based on the acute clinical presentation and CSF results, herpetic encephalitis was initially suspected. However, the symmetric limbic pathology that was shown on MRI questioned the diagnosis, since herpetic lesions mainly progress unilaterally across the whole temporal lobe. Moreover, serologic and PCR studies failed to detect any evidence of HSV or other herpesvirus infection and despite treatment with intravenous acyclovir, the patient had a rapidly progressive cognitive decline. Bilateral lesions in medial temporal lobes could be also attributed to HHV-6 infection that has rarely been reported after allogeneic hematopoietic stem cell transplantation [4]. However, PCR was negative in our patient and excluded this diagnosis.

A second CSF analysis revealed oligoclonal bands indicating humoral autoimmunity against the CNS. Antibodies against Hu were detected and an extensive screening for underlying neoplasia revealed a large cell neuroendocrine carcinoma of the lung. The association of LE with latent neuroendocrine cancer and antibodies to Hu set the diagnosis of anti-Hu PLE [5].

PLE is an immune mediated disorder, the best evidence for which comes from the demonstration of antineuronal antibodies in patient's CSF and serum. These antibodies react with neuronal proteins that are usually expressed by the patient's tumour, and their detection is the basis of useful diagnostic tests. PLE usually follows a more protracted clinical course characterized by a gradual evolution of symptoms within weeks or months; thus, the acute onset of encephalopathy with fever and confusion in our patient, mimicking acute viral encephalitis, constitutes an uncommon presentation.

Few cases of acute LE that are not associated with herpetic infection have been recently reported in the literature. In Japan, cases of acute encephalitis with a clinical presentation similar with HSV encephalitis have been reported. These cases did not demonstrate evidence of HSV infection and have been named “nonherpetic acute limbic encephalitis” as a possible new subgroup of LE of unknown aetiology [6]. However, among these cases described in Japan, there is a subgroup of severe but often reversible encephalitis that predominantly affects young women, called “juvenile acute nonherpetic encephalitis”. In many of these patients an antibody against NR1/NR2 heteromers of the NMDA receptor was detected; thus, an NMDAR encephalitis was diagnosed [7]. NMDAR encephalitis usually affects young women and may initially present as a viral-like illness. About 50% of these patients have an underlying tumor, usually a cystic ovarian teratoma [1, 8]. In addition, voltage-gated potassium channel complex antibodies limbic encephalitis (mainly LGI1) may present with acute to subacute onset memory loss, confusion, mediotemporal lobe seizures, agitation, and other psychiatric features, evolving over several days or weeks [8].

Patients with anti-Hu antibodies develop LE that may progress to widespread encephalomyelitis. Most patients are smokers and the associated tumor is a lung tumor in 85% of patients (typically a small-cell lung carcinoma), while in 15% there is an extrathoracic neoplasm including prostate, gastrointestinal, breast, bladder, pancreas, and ovary. However, approximately 50% of patients with these tumors and LE have no antibodies to Hu and better prognosis than patients with anti-Hu antibodies [1–3].

According to previous published series of anti-Hu PLE, there has not been reported acute onset of symptoms mimicking viral encephalitis [2, 3]. The evolution of symptoms was subacute reaching a plateau within 6 months in 95% of patients, while it was chronic and mild resulting in slow progression over years in 5% [3].

Up to date, there is only a single case report in the literature of PLE with small cell lung cancer (both diagnosed at necropsy) that presented as acute viral encephalitis with headache, myalgia, and fever [9]. Two cases of PLE coexisting with HSE have also been reported. The first one with lung adenocarcinoma and antibodies to Ma2 that developed HSV encephalitis confirmed by CSF PCR after 6 weeks [10]. The second with antibodies to Hu (no identifiable neoplasm) that a HSV encephalitis was confirmed by postmortem immunocytochemistry and positive PCR performed on temporal tissue extracts [11].

In conclusion, anti-Hu PLE must be encompassed in the differential diagnosis of acute encephalitis and is of great clinical significance, given that this rare disorder may respond well to immunosuppressive and antineoplastic treatment.
